# Pan American Health Organization/World Health Organization Collaborating Centers in Nursing and Midwifery in Haiti

**DOI:** 10.26633/RPSP.2019.30

**Published:** 2019-03-15

**Authors:** Nancy Ambrose Gallagher, Megan Eagle, Norma Sarkar, Silvia Cassiani, Jody Lori

**Affiliations:** 1 University of Michigan University of Michigan School of Nursing Ann ArborMichigan United States of America School of Nursing, University of Michigan, Ann Arbor, Michigan, United States of America.; 2 Pan American Health Organization Pan American Health Organization Washington, D.C. United States of America Pan American Health Organization, Washington, D.C., United States of America.

**Keywords:** Pan American Health Organization, nursing, midwifery, universal health coverage, Haiti, Organización Panamericana de la Salud, enfermería, partería, cobertura universal de salud, Haití, Organização Pan-Americana da Saúde, enfermagem, tocologia, cobertura universal de saúde, Haiti

## Abstract

**Objective.:**

To describe partnerships that Pan American Health Organization/World Health Organization (PAHO/WHO) Collaborating Centers in Nursing and Midwifery have in Haiti, and their contribution to promoting universal health coverage in that country.

**Methods.:**

In 2017, semistructured interviews were conducted by telephone or email to update the status of activities and collaborations that were mentioned in a 2016 report (which covered efforts prior to early 2016) by the office of the Regional Advisor on Nursing and Allied Health Personnel at PAHO/WHO. Using that information, two of the authors categorized the Collaborating Center activities into focal areas.

**Results.:**

Six of the nine Collaborating Centers mentioned in the 2016 PAHO/WHO report participated in the 2017 semistructured interviews. The five focal areas identified were: 1) direct care/primary health care, 2) research, 3) workforce development, 4) curriculum development, and 5) shared educational activities.

**Conclusions.:**

Current PAHO/WHO Nursing and Midwifery Collaborating Center partnerships in Haiti support universal health access and coverage through direct provision of care with ongoing Haiti-based clinics; research in topics relevant to Haitian partners; assistance with continuing education for nurses; and shared educational activities. These efforts are enhanced through partnerships with Haitian organizations and the Ministry of Public Health and Population. Coordination among PAHO/WHO Collaborating Centers could augment individual schools’ efforts to assist health providers and institutions in Haiti to improve health outcomes and support universal health coverage.

## RESUMEN

**Objetivo.:**

Describir las alianzas que tienen los centros colaboradores de la Organización Panamericana de la Salud/Organización Mundial de la Salud (OPS/OMS) en enfermería y partería en Haití y su contribución a la promoción de la cobertura universal de salud en ese país.

**Métodos.:**

En el 2017, se llevaron a cabo entrevistas semiestructuradas por teléfono y por correo electrónico para actualizar el estado de las actividades y colaboraciones mencionadas en un informe del 2016 (que abarcaba esfuerzos previos a los primeros meses del 2016) realizado por la oficina del asesor regional en materia de enfermería y personal paramédico de la OPS/OMS. Con base en esa información, dos de los autores clasificaron las actividades de los centros colaboradores en áreas focales.

**Resultados.:**

Seis de los nueve centros colaboradores mencionados en el informe de la OPS/OMS del 2016 participaron en las entrevistas semiestructuradas del 2017. Las cinco áreas focales establecidas fueron: 1) atención directa/atención primaria de salud, 2) investigación, 3) desarrollo de la fuerza laboral, 4) elaboración de programas de estudios y 5) actividades educativas compartidas.

**Conclusiones.:**

Las alianzas actuales de los centros colaboradores de la OPS/OMS en enfermería y partería en Haití brindan apoyo al acceso y la cobertura universales de salud por medio de la prestación directa de atención en los consultorios permanentes establecidos en Haití, investigan sobre temas de importancia para los asociados haitianos, ofrecen asistencia con respecto a la educación continua para enfermeras y realizan actividades educativas compartidas. Estos esfuerzos se optimizan por medio de las alianzas con organizaciones haitianas y el Ministerio de Salud Pública y Población. La coordinación entre los centros colaboradores de la OPS/OMS podría potenciar los esfuerzos individuales de las facultades para ayudar a los prestadores y las instituciones de salud de Haití a mejorar los resultados en materia de salud y brindar apoyo a la cobertura universal de salud.

## RESUMO

**Objetivos.:**

Descrever as parcerias existentes com os Centros Colaboradores da Organização Pan-Americana da Saúde/Organização Mundial da Saúde (OPAS/OMS) para Enfermagem e Obstetrícia no Haiti e como elas contribuem para promover a cobertura universal de saúde no país.

**Métodos.:**

Em 2017, foi feito um levantamento por meio de entrevistas semiestruturadas por telefone ou email para atualizar a situação das atividades e trabalhos de colaboração descritos em um informe de 2016 (referente aos esforços anteriores a 2016), sob a coordenação do escritório do Assessor Regional de Enfermagem e Pessoal de Saúde Associado da OPAS/OMS. A partir destas informações, dois coordenadores categorizaram as atividades dos centros colaboradores em domínios prioritários.

**Resultados.:**

Seis dos nove centros colaboradores citados no informe da OPAS/OMS de 2016 participaram das entrevistas semiestruturadas de 2017. Os cinco domínios prioritários identificados foram: 1) atenção direta/atenção primária à saúde, 2) pesquisa, 3) aperfeiçoamento do pessoal, 4) desenvolvimento curricular e 5) atividades educacionais conjuntas.

**Conclusões.:**

As atuais parcerias dos Centros Colaboradores para Enfermagem e Obstetrícia da OPAS/PMS no Haiti promovem o acesso universal à saúde e a cobertura universal de saúde com a prestação direta de assistência em ambulatórios permanentes no Haiti, pesquisas em tópicos relevantes aos parceiros no Haiti, auxílio com educação continuada para o pessoal de enfermagem e atividades educacionais conjuntas. Esses esforços são fortalecidos com parcerias entre as organizações e o Ministério de Saúde Pública e População do Haiti. Com a coordenação entre os centros colaboradores da OPAS/OMS, poderiam ser ampliados os esforços individuais das escolas a fim de ajudar os prestadores de serviços e instituições de saúde no Haiti a melhorar os desfechos de saúde e promover a cobertura universal de saúde.

The United Nations, in its Sustainable Development Goal 3 (“ensure healthy lives and promote well-being for all at all ages”), recognizes that a healthy population is a critical component of economic and social development (1). While health is influenced by many factors (including adequate nutrition, clean water, sanitation conditions, and natural disasters), access to quality health care is also critically important to improving health in low-resource settings. A key target of Sustainable Development Goal 3 is universal health coverage—including health financing and the recruitment, development, training, and retention of nurses and other health care providers, in both developing and least-developed countries, such as Haiti.

Haiti has been described by the author Edwidge Danticat as a country of beautiful art, beautiful music, and resilient people (2). Despite its strengths, Haiti is a country with an ongoing need for support in developing its health care infrastructure. The poorest country in the Western Hemisphere (3), Haiti has faced a succession of natural disasters, including multiple major hurricanes and the country’s worst earthquake in 200 years. These events have impacted the country’s health both directly and indirectly, through its health infrastructure and capacity for educating nurses and midwives. More than 200 000 casualties resulted from the 2010 earthquake, including most of the sophomore class of nursing students in the public nursing school in Port-au-Prince, as well as many faculty (3, 4). In addition, many educational facilities and public buildings were destroyed, including the nursing school itself, the Ministry of Health, and multiple hospitals and health centers (3).

The Pan American Health Organization/World Health Organization (PAHO/WHO) Collaborating Centers are part of an international network that conducts activities to support WHO’s strategic plans (5). The Nursing and Midwifery Collaborating Centers are designed to serve as a resource for knowledge exchange, capacity-building, and health system development in the Americas (6). This role may be particularly critical in Haiti, given its history of natural disasters and poverty. The purpose of this paper is to describe the activities and partnerships of the PAHO/WHO Nursing and Midwifery Collaborating Centers in Haiti and their contribution to the promotion of universal health coverage.

## HEALTH, HEALTH CARE, AND NURSING IN HAITI

Despite the challenges it has faced, Haiti has made health gains in recent decades, including a steady decline in the crude death rate (7) and in mortality among children under 5 years of age (8). The national response to HIV has intensified, and the incidence of the disease has declined (7), as has mortality among those with HIV (9). However, maternal and neonatal mortality remain among the highest in the world (10, 11) and child and maternal malnutrition are one of the leading risk factors driving death and disability in the country (12). While diarrheal diseases remain the leading cause of lost disability-adjusted life years in Haiti, cardiovascular disease has emerged as the second leading cause, followed by HIV/AIDS, tuberculosis, and noncommunicable diseases such as neoplasms and diabetes. Ischemic heart disease and cerebrovascular disease overtook HIV/AIDS as the leading cause of death in the years between 2005 and 2015. Other chronic diseases, such as diabetes and hypertension, have also increased in prevalence and rank order as a cause of death (12). Additionally, more than 35% of the population is now overweight (13). These changes highlight the emerging importance of educating health care providers, including nurses, to design and implement health interventions to manage and prevent chronic diseases in the country, as well as strengthening the settings that support these interventions.

Primary health care is the cornerstone of a functional health system (8) and is a global priority (14). Following the 2010 earthquake in Haiti, the Ministry of Public Health and Population developed a new community health care model based on primary care and an integrated network of health services. Services at three levels include community hospitals and health centers, 10 departmental hospitals, and university hospitals and specialty centers. In the community health centers, teams of one physician and two nurses supervise community health workers who conduct home visits and provide disease prevention and health promotion services (7). A 2013 national census of health centers in Haiti indicated that 87% of health facilities were primary care facilities (8), and 91% of the population live within 5 kilometers of a primary care facility. However, urban residents have access to higher quality primary care (8).

In addition to primary health care, a functioning public health infrastructure is critical for maintaining and improving health and well-being (15). While gaps in Haiti’s public health system complicated its ability to respond to the earthquake and the cholera epidemic that followed, important improvements have occurred (15, 16). In the aftermath of the 2010 earthquake, surviving public health leaders built on strategies from their successful HIV program to improve access to primary health care for pregnant women and children, including improving testing for sexually transmitted disease and boosting childhood vaccination rates (16). Enhanced surveillance systems put in place during the cholera epidemic have helped to reduce the impact of subsequent outbreaks by providing rapid communication with clinicians across the country about recommended treatment regimens based on susceptibility testing (16).

In addition to infrastructure, a qualified work force is essential to an effective health system. Nurses are critical to achieving universal health coverage and access to primary health care. WHO recommends at least 4.45 nurses, physicians, and midwives per 1 000 people (17). In Haiti there are 1.4 physicians and 1.8 nurses per 10 000 people in the public sector, with 1 physician and 2.1 nurses per 10 000 people in the private sector (7). This is one-fourth of the world’s average nurse-to-population ratio (3) and the lowest in the Americas (18). In addition, there is only one midwife for every 50 000 inhabitants (19), and the majority of births in the country take place at home and are not attended by a skilled birth attendant or midwife (10). Approximately half of the nurses leave Haiti within five years of graduation, due to poor working conditions, lack of incentives, and limited employment opportunities (3), highlighting the need for improved workforce development and retention. The average pay for nurses in Haiti is US$ 400–500 per month, with many nurses experiencing high workloads and often working months without pay (20). Rural areas are especially underserved by nurses. Seventy percent of nurses work in Port-au-Prince, the capital of Haiti, where only about one-third of the country’s residents live. Outside Port-au-Prince, most small hospitals have only one or two qualified nurses employed (3).

Nursing education in Haiti has the potential to improve health care delivery and universal access and coverage through improved nurse retention and performance. However, the increasing burden of chronic disease and emphasis on primary and community-based health care will require major changes in nursing practice and education (3). Haiti’s five leading nursing schools graduate approximately 200 to 300 nurses a year. Currently, most prelicensure nursing schools are diploma programs with curriculums that have changed little in recent decades. Few schools in Haiti offer a bachelor’s degree in nursing, and most training is focused on inpatient hospital care (3). Many nurses are unprepared by their hospital-based training for community-based experiences, and would benefit from opportunities for continuing education in public health and primary health care (3).

In addition, many nurses in Haiti find themselves taking on advanced roles in their practice when advanced practitioners are unavailable, but few options exist for a regulated role as an advanced practice nurse (3). Haiti’s only school of midwifery at the time of the earthquake was damaged, with an interruption in the educational program until the school was moved to a temporary shelter (19). The school reopened in 2013 (19). In addition, the first family nurse practitioner (FNP) program in Haiti was started at the Faculté des Sciences Infirmières de l’Université Episcopale d’Haïti (FSIL), and 16 FNPs graduated with a master’s degree in November 2017. This first program developed as a partnership between the nonprofit Promoting Health in Haiti (PHH) and FSIL. The FNP program now continues solely as a part of FSIL (personal communication, J. Pohl, 21 April 2018).

## PAHO/WHO COLLABORATING CENTERS IN NURSING AND MIDWIFERY IN HAITI

WHO relies on more than 46 Collaborating Center in Nursing and Midwifery institutions around the world to carry out its mission of improving health, especially among disadvantaged populations (21). The PAHO/WHO Collaborating Centers in Nursing and Midwifery have a strong presence in Haiti, with 9 Collaborating Centers currently working toward the advancement of nursing in Haiti (21). These 9 centers are based at the Universidad de Chile, University of Miami, University of Pennsylvania, University of Illinois-Chicago, University of Michigan, Johns Hopkins University, McMaster University, University of Maryland, and Universidade de São Paulo.

## METHODS

### Study design

We used a qualitative, descriptive design to portray activities in Haiti of PAHO/WHO Collaborating Centers in Nursing and Midwifery. We reviewed a 2016 report by the office of the Regional Advisor on Nursing and Allied Health Personnel at PAHO/WHO that outlined nursing and midwifery collaborations of nine Collaborating Centers that had reported engagement in Haiti between 2000 and early 2016 (22). (While most of those activities were carried out after the 2010 earthquake, some had taken place before that.) Nursing faculty who were identified in the 2016 PAHO/WHO report as being involved in PAHO/WHO Collaborating Center activities were contacted by email to set up a telephone interview.

The sample for our research included six Collaborating Centers that responded to our request for updated information on activities in Haiti since the 2016 PAHO/WHO report. A semistructured interview guide was used to update the original 2016 PAHO/WHO report. Use of a semistructured interview allowed for consistent information to be obtained from each of the six participating Collaborating Centers (24).

Two journal articles (published in 2013 and 2014) also reported on Collaborating Center work in Haiti, and we reviewed those publications for historical context and additional details about those efforts (10, 23).

The project was deemed to be exempt from ongoing review by the Institutional Review Board at the University of Michigan.

The semistructured interviews were conducted in 2017 and explored the status of previous and current activities in Haiti. Questions included identifying specific activities engaged in, as well as local collaborating organizations. All but one of the six responding Collaborating Centers chose to respond by email; faculty at the remaining center responded in a telephone interview. The single telephone interview was not recorded. The telephone interview was conducted with two faculty members; all other communications were with single individuals. In some cases, a second or third phone call or email communication was initiated if a center did not initially respond or if additional information or clarification was needed.

Three of the nine Collaborating Centers mentioned in the 2016 PAHO/WHO report did not respond, and information on any of their recent activities is not included in this article. However, information on previous activities of those three Collaborating Centers was included, based on information from the 2016 PAHO/WHO report (22).

### Data analysis

Written notes from the semistructured interviews were coded for Collaborating Center activities by the first author (NAG), followed by categorization into focal areas encompassing the activities. The themes and coding were then discussed by the first author and the last author (JL), and agreement was reached on the relevant focal areas, as shown in Table 1.

**TABLE 1:** Focal areas and related activities of PAHO/WHO Collaborating Centers in Nursing and Midwifery in Haiti, 2000-2017^a^

PAHO/WHO Collaborating Center resource categories	Collaborating Center focal areas and related activities
Health systems development	Direct care Faculty partnered with Mountain Top Ministries to assist with provision of primary care, social work, and pharmacy services in mobile clinics run by Haitian nurses and doctors in Thomonde Faculty and advanced practice registered nurse (APRN) students provide medical supplies and health care professionals for rural primary care clinic run by Haitian health care providers four times a year (January, March, August, and October) for one week Faculty provided midwifery care in outlying clinic with Haitian students^b^ Faculty collaborated with Médecins Sans Frontières and Partners In Health to provide care for cholera patients^b^ One faculty member and APRN students have provided primary care at clinics and schools for 10 years^b^
Capacity development	Workforce development Developed mental health training manual and program after 2010 earthquake. Workbooks and training manuals were produced in Haitian Creole, French, and English. Training now being conducted by Haitian nurses. Met with Ministry of Health and Les Cayes School of Nursing to develop continuing education for nurses related to maintaining patient safety. Provided a weeklong educational session for rural primary care clinic four times a year (January, March, August, and October) Trained 300 community health workers to disseminate information on prevention of sepsis and to advocate for health promotion Worked to bring midwives into clinics and hospitals Faculty and graduate students spent two weeks to one month on a U.S Centers for Disease Control and Prevention project to train health care workers to be educators in infectious disease control and HIV prevention Faculty attended and made three presentations at first annual Haitian midwifery conference^b^ and assisted with its evaluation^b^ Participated with Formation des professionnels de la santé to strengthen health services by increasing the number and quality of services provided by primary front-line service providers, via basic and continuing education for nurses, midwives, and lab technicians, with a particular emphasis on maternal and child health^b^
Curriculum development	Curriculum development Worked on program for direct entrance to School of Midwifery Conducted a needs assessment and proposal to increase maternal and child health curriculum and capacity Conducted workshops given on pedagogy of teaching Enhanced environmental health curriculum and validated first module of environmental health course Provided classes to Midwives for Haiti for midwifery education^a^ Midwifery faculty mentored students and faculty on how to develop an effective precepting relationship while providing antenatal, delivery, and postpartum care^b^ Conducted simulation training with Midwives for Haiti faculty to evaluate students in postpartum hemorrhage management with available resources. Prepared material in French and Haitian Creole.^b^ Worked with Midwives for Haiti faculty on graduate-level course on maternal and child health in the Americas^b^
Knowledge exchange	Shared educational activities In the fall semester since 2012, for community health nursing courses, videoconferencing has taken place between FSIL in Haiti and senior-level students in a U.S. school of nursing An electronic community of practice (eCoP) was designed and implemented at FSIL in 2013, and a university grant used to improve their Internet capability. Undergraduate students conduct community health projects related to public health at a rural clinic established by a faculty member^b^
	Research Faculty work with Institute of Human Virology, the University of Notre Dame in Port-au-Prince, and local health care providers on infection control and HIV prevention Faculty and advanced practice students partner with Global Health Action to conduct data collection on umbilical cord care and sepsis prevention Faculty and students spent one week in data collection (interviews, surveys, and focus groups) and research on evidence-based care^b^ Two faculty are consultants on research with women and girls in post-disaster settings^b^

***Source*:** Prepared by the authors, based on the study results.

a Most of the PAHO/WHO Collaborating Center activities were carried out after the 2010 earthquake in Haiti, but a small number had taken place before that.

b University did not respond to the 2017 interview requests, so data is included from 2016 PAHO/WHO report (22).

## RESULTS

Six of the nine Collaborating Centers in the 2016 PAHO/WHO report responded. Five focal areas consistent with the goals of the PAHO/WHO Collaborating Centers (health system and capacity development, and knowledge exchange) were identified: 1) direct care/primary health care, 2) research, 3) workforce education and development, 4) curriculum development, and 5) shared educational activities between students in Haiti and in Collaborating Center educational institutions.

### Health systems development: direct care/primary health care

Four PAHO/WHO Collaborating Center partnerships with Haitian health care providers supported the direct provision of primary health care services. Two Collaborating Centers provided updated information. One assists Haitian nurses and physicians in mobile clinics in the commune of Thomonde that provide primary care, social work, and pharmacy services. A second provides health care professionals and medical supplies for one week every quarter in support of a year-round clinic run by Haitian providers. A third (nonresponding) Collaborating Center has provided midwifery services in a rural clinic with Haitian students. This Collaborating Center also worked together with two nongovernmental organizations (NGOs) (Médecins Sans Frontières (an international humanitarian organization providing medical services) and Partners In Health (the largest nonprofit health care provider in Haiti)) to provide care for patients with cholera (25, 26). A fourth Collaborating Center has sent a faculty member and nurse practitioner students each year to provide care in clinics and schools (22).

### Capacity development: workforce education and development

Seven Collaborating Centers have worked with Haitian colleagues in workforce development and continuing education (five of these seven responded to our interview request). After the earthquake in 2010, one Collaborating Center developed mental health training and workbooks in Haitian Creole, French, and English on the diagnosis and treatment of trauma. More than 100 health workers participated in this mental health training to increase local mental health care capacity (23). The same Collaborating Center worked with the Les Cayes School of Nursing and the Haiti Ministry of Health to develop continuing education for nurses on patient safety.

In their quarterly work assisting with direct provision of primary care services in the commune of Petit-Goâve, Haiti, a second Collaborating Center also provides educational sessions for local health care professionals. A third Collaborating Center trained 300 community health workers to disseminate materials related to health promotion and the prevention of sepsis. A fourth worked to increase the number of midwives in hospitals and clinics. A fifth Collaborating Center has evaluated occupational health and safety among health care workers in the past, and has trained over 70 nurses in infectious disease.

One university that did not respond to our information request attended and presented at Haiti’s first midwifery conference, and assisted with its evaluation. These faculty members have also worked with Midwives for Haiti (a private NGO that provides training for midwives in Haiti) on a train-the-trainer program (27). A second nonresponding Collaborating Center partnered with Formation des professionnels de la santé (a consortium of Canadian health and professional institutions aimed at strengthening health services by increasing the number and quality of services provided by front-line service providers in Haiti) in providing continuing education for nurses, midwives, and laboratory technicians, with a particular emphasis on maternal and child health (22).

### Capacity development: curriculum development

Four Collaborating Centers have worked on curriculum development, particularly for midwives; three responded to the interview request. One Collaborating Center has worked on a program for direct entry to the School of Midwifery and has assisted with opening a second midwifery program. A second Collaborating Center has worked to improve capacity in midwifery and family planning with a five-year project for curriculum development. A third has provided education on teaching pedagogy and has expanded nursing education on environmental health. Finally, a fourth Collaborating Center has assisted Midwives for Haiti with course development using simulation, traditional materials in French and Haitian Creole, and mentorship on developing preceptor skills (22).

### Knowledge exchange: shared educational activities

Two Collaborating Centers have engaged in shared educational activities. Faculty at one have had a long-standing relationship with the Faculté des Sciences Infirmières de l’Université Episcopale d’Haïti (FSIL), the first four-year baccalaureate nursing school in Haiti. This Collaborating Center has facilitated videoconferencing between the two institutions since 2013, providing regular communication between senior-level nursing students studying community health at both schools (28). Students are encouraged to use the Knowledge Gateway electronic community of practice (eCoP), which is available through WHO, to increase the exchange of knowledge, experiences, and perspectives (28). In addition, digital subscriber line (DSL) cable was laid at FSIL to improve the students’ Internet access (P. Abbott, personal communication, 24 September 2014). Students at both the Collaborating Center and FSIL describe exposure to other cultures and health care delivery systems as benefits of the videoconferencing experience, and note both the differences and the similarities in social determinants of health between the two countries (29). Undergraduate community health nursing students from a second (nonresponding) Collaborating Center have conducted community health projects in Haiti at a clinic established there by a faculty member.

### Knowledge exchange: research

Four Collaborating Centers, two of which responded to the interview request, have conducted research on health problems important to Haiti. One has partnered with the Institute of Human Virology in Haiti to work with local health care providers on infection control, HIV prevention, and occupational health issues, while faculty and advanced practice students from another Collaborating Center, partnering with Global Health Action (an NGO conducting health and development programs in Haiti), have assisted in research and data collection related to sepsis and postpartum care of the umbilical cord (30). A third (nonresponding) Collaborating Center also has assisted with data collection and research on health issues related to maternal and child health, while faculty from a fourth reported serving as consultants on research on women and girls in a post-disaster setting (22).

## DISCUSSION

This article has described PAHO/WHO Collaborating Center in Nursing and Midwifery activities in Haiti. Five focal areas of Collaborating Center activities emerged from the analysis: direct care/primary care, workforce development, curriculum development, shared educational activities, and research.

Universal access to health and health coverage strengthen health care delivery systems and improve health outcomes, particularly for disadvantaged populations (31). However, in low- and middle-income countries such as Haiti, the health burden of HIV and noncommunicable diseases, coupled with a shortage of health care workers, threatens the sustainability of these health systems and the achievement of universal health care coverage (32). While each of the activities described above support the overall mission of PAHO/WHO, coordination of these efforts could increase the impact and sustainability of these activities in support of universal health access and coverage.

Haiti has built on successful programs, such as its model HIV program, to build primary care, maternal and child care, and community/public health in the country. However, it is clear that gaps remain in both provision of primary health care services and in the availability of health care providers, particularly in rural regions (3). The engagement of the PAHO/WHO Collaborating Centers in Nursing and Midwifery, and continued partnerships and coordination with ongoing primary care providers, has the potential to assist Haitian providers in increasing universal access and health coverage across the country through health system and capacity development. Several Collaborating Centers provide direct care, supplementing ongoing Haiti-based primary health care clinics. Others assist with continuing education of current nurses, or with the education of nurses at the undergraduate or graduate level. This is especially true for the education of midwives, which is a critical need in a country where the rate of maternal and infant deaths is the highest in the Western Hemisphere. Haiti’s Ministry of Public Health and Population has identified child health as one of the country’s top health issues (33). Additional communication and coordination among the Collaborating Centers and health care professionals and systems in Haiti could increase efforts targeted at those various high-priority needs.

Given the high burden of infectious and parasitical diseases as well as nutritional conditions, Haiti needs a strong public health presence with mid-level community health workers and with professional nurses who are able to supervise those community health workers (3). PAHO/WHO Collaborating Center efforts in workforce development can help to provide continuing education related to public and community health for currently practicing health care providers. These projects, some in collaboration with Haiti’s Ministry of Public Health and Population, have included curriculum related to patient safety, primary care, environmental and occupational health, and midwifery education. Sharing resources and personnel in research on areas of importance to Haiti, such as prevention of sepsis, can also enhance these efforts.

PAHO/WHO Collaborating Center relationships with Haitian universities at the undergraduate level support those universities’ work in increasing the number of nurses available at both the generalist and advanced practice levels. Given the current educational system focus on hospital-based care, support of baccalaureate nursing education that extends beyond a focus on inpatient care to primary and community-level care is critical (31). In association with the Government of Haiti, PAHO/WHO Collaborating Centers could assist nursing programs in Haiti in training nurses in community and population health. Increasing the number of graduates from these schools of nursing will also boost the number of nurses who can move into an advanced practice role (31). At FSIL, for example, most of the nurses who graduate from the baccalaureate program remain in Haiti to practice (34). In addition, health system services and university partners could work with the Government in further supporting incentives and retention efforts for current nurses.

Finally, PAHO/WHO has recommended the inclusion of advanced practice nurses such as nurse practitioners and midwives as part of a strategy to increase the primary health care work force in Latin American and Caribbean countries (3, 32). The involvement of several Collaborating Centers in midwifery education supports this strategy. Further development of partnerships among nurses, midwives, and lay birth attendants, as well as training for the birth attendants on morbidity-mortality prevention, may further support Haitian efforts to improve maternal and child health outcomes (10).

### Limitations

Limitations of this work include unavailable information from three Collaborating Centers that did not respond to interview requests. Several efforts were made to contact all the Collaborating Centers, and it may be that these nonrespondents’ work in Haiti has been concluded. However, previous work cited in the PAHO/WHO 2016 report was included in order to acknowledge their important work in Haiti. In addition, information about funding as well as the context of the Collaborating Center activities within the larger Haitian and global health systems was unavailable. Future work should examine these areas in more detail, as well as the potential role of the Collaborating Centers in influencing health policy in Haiti that could affect universal health coverage.

## Conclusions

Haiti has made substantial improvements in health and health care in recent decades (7, 8). However, there are still critical health concerns that could be mitigated by addressing universal health coverage and social determinants of health. These challenges include high maternal and neonatal mortality rates, malnutrition, and an increasing prevalence of chronic disease and obesity (10-12). Current PAHO/WHO Collaborating Center in Nursing and Midwifery partnerships in Haiti support universal health access and coverage through a variety of initiatives. Among these are the direct provision of primary health care in concert with ongoing Haiti-based clinics; research in topics relevant to Haitian partners; and assistance with continuing education for practicing nurses and with undergraduate and graduate education, particularly related to primary and community care and maternal and child health. These efforts are enhanced through continued partnerships with Haitian organizations and the Ministry of Public Health and Population. Future work by these Collaborating Centers may include coordination with other PAHO/WHO Collaborating Centers and evaluation of the impact of these activities on health outcomes. These efforts could augment individual schools’ efforts to assist health professionals and institutions in Haiti to advance health outcomes and to continue to support universal access to health and universal health coverage.

## Author contributions.

All the authors (NAG, ME, NS, SC, and JL) conceived of the idea for the manuscript. NAG helped to collect the data. NAG and JL analyzed the data and interpreted the results. NAG wrote the paper, and all the authors (NAG, ME, NS, SC, and JL) reviewed and approved the final version.
